# Walking in fully immersive virtual environments: an evaluation of potential adverse effects in older adults and individuals with Parkinson’s disease

**DOI:** 10.1186/s12984-017-0225-2

**Published:** 2017-02-21

**Authors:** Aram Kim, Nora Darakjian, James M. Finley

**Affiliations:** Division of Biokinesiology and Physical Therapy, University of Southern California, 1540 E. Alcazar St, CHP 155, Los Angeles, CA 90033 USA

**Keywords:** Virtual reality, Parkinson’s disease, Head-mounted display, Gait, Simulator sickness

## Abstract

**Background:**

Virtual reality (VR) has recently been explored as a tool for neurorehabilitation to enable individuals with Parkinson’s disease (PD) to practice challenging skills in a safe environment. Current technological advances have enabled the use of affordable, fully immersive head-mounted displays (HMDs) for potential therapeutic applications. However, while previous studies have used HMDs in individuals with PD, these were only used for short bouts of walking. Clinical applications of VR for gait training would likely involve an extended exposure to the virtual environment, which has the potential to cause individuals with PD to experience simulator-related adverse effects due to their age or pathology. Thus, our objective was to evaluate the safety of using an HMD for longer bouts of walking in fully immersive VR for older adults and individuals with PD.

**Methods:**

Thirty-three participants (11 healthy young, 11 healthy older adults, and 11 individuals with PD) were recruited for this study. Participants walked for 20 min while viewing a virtual city scene through an HMD (Oculus Rift DK2). Safety was evaluated using the mini-BESTest, measures of center of pressure (CoP) excursion, and questionnaires addressing symptoms of simulator sickness (SSQ) and measures of stress and arousal.

**Results:**

Most participants successfully completed all trials without any discomfort. There were no significant changes for any of our groups in symptoms of simulator sickness or measures of static and dynamic balance after exposure to the virtual environment. Surprisingly, measures of stress decreased in all groups while the PD group also increased the level of arousal after exposure.

**Conclusions:**

Older adults and individuals with PD were able to successfully use immersive VR during walking without adverse effects. This provides systematic evidence supporting the safety of immersive VR for gait training in these populations.

## Background

Parkinson’s disease (PD) is a progressive neurodegenerative disorder resulting from a loss of dopaminergic cells in the substantia nigra, and affects over 1 million individuals over age 65 in the United States and over 7 million worldwide [1, 2]. Common symptoms of PD are impairments in balance and gait, rigidity, tremor and bradykinesia. Although dopamine replacement therapy (DRT) is commonly prescribed to individuals with PD, pharmacological interventions, especially in the late course of PD, become inadequate resulting in a progressive deterioration in mobility and activities of daily living [3]. In addition, some of the gait deficits such as stride time variability are related to the non-dopaminergic lesions [3, 4]. Increasing evidence supports that task-specific, goal-based motor skill training promotes neuroplasticity and reduces motor impairments in individuals with PD [4–8]. Although task-specific training is often used in the clinic, it may not be possible to configure the clinical setting to recreate the environmental contexts and challenges that patients might experience in the real world.

In order to address these issues, one recent solution for providing task-specific gait training in environments that mimic the real world is through the use of virtual reality (VR) interventions [9–14]. VR can help to overcome some of the space and resource limitations found in traditional clinics while also allowing patients to repeat the practice of advanced gait skills necessary for community ambulation [15]. To date, a small number of studies have investigated gait training for individuals with PD using non-immersive VR [9, 13, 16, 17]. However, the coupling between perception and action in non-immersive VR can be quite different than in the real world due to an indirect mapping from physical movement to movement of one’s representation in the virtual environment. To achieve a more natural experience, immersive VR simulations are often generated using a head-mounted display (HMD) [11, 12, 14] or room-scale displays that surround the user such as the CAVE [18, 19]. In both cases, immersion and presence are heightened when the motion of visual field is properly linked with the motion of the head.

Different viewpoints and modes of navigation can be provided to modify the user’s experience in VR. Viewpoints are typically separated into first and third person perspectives [20]. The first person perspective allows the user to perceive the simulation through the eyes of a character, whereas the third person perspective forces the user to observe their character from a distance. Moreover, two types of navigation are also possible in VR: egocentric and exocentric navigation [21]. In egocentric navigation, the user’s viewpoint is surrounded by the environment while in exocentric navigation, the user looks into the environment from outside. Generally, a first person viewpoint with egocentric navigation provides more immersion [20] and a more natural visual experience [22] in VR as it allows the user to be part of the simulation, rather than an observer. This allows users to experience a higher degree of presence than non-immersive environments [23]. Presence is defined as “the subjective experience of being in one place or environment, even when one is physically situated in another” [24]. High presence in immersive VR for rehabilitation has several benefits [25]. First, users may “forget” that they are in a training situation. This may lead to the use of more natural motor patterns and thereby improve the ecological validity of training. Second, situations that usually cannot be evaluated in the clinic, such as walking at night or in inclement weather, can be simulated. Third, users are able to view the virtual world in a manner that provides more natural sensory information [26]. Thus, high presence in immersive VR has been suggested to be more effective training approach than non-immersive VR as it allows provides the user with a more realistic sensorimotor experience.

Despite these benefits, there may be adverse effects of experiencing VR that might preclude its use in the clinic. These adverse responses are commonly termed simulator sickness [27]. Regan and Price explored the effects of a 20 min-exposure to a virtual environment through an HMD and found that 61% of healthy young participants experienced some degree of simulator sickness symptoms [28]. Sharples and colleagues reported that VR exposure induced more simulator sickness symptoms in healthy young adults using an HMD than using non-immersive devices such as a desktop or a projector [29]. In contrast, a recent study showed that healthy young adults experience no or minor simulator sickness during neck motion-controlled VR tasks [30]. This discrepancy is most likely due to recent advances in technology that have resulted in inexpensive, high-performing systems that can minimize adverse responses such as simulator sickness [31].

Moreover, there are concerns that populations such as older adults or individuals with neurological disorders may be more likely to have adverse responses to fully immersive experiences than healthy, young individuals. There are two common theories to explain the occurrence of simulator sickness: (1) sensory conflict theory and (2) postural instability theory [32]. Sensory conflict theory suggests that simulator sickness arises from conflicts between different senses that cause incompatibility with stored expectations [33, 34]. For example, conflicts can occur between the motion that a person perceives through optic flow and the motion detected by the vestibular and somatosensory systems. These mismatches may be even more pronounced in older adults due to sensory processing deteriorations [35] and in individuals with PD due to impairments in sensory processing and integration [36–38]. These deficits in sensory processing and integration in individuals with PD could potentially generate conflicts between visual and somatosensory information in a virtual environment, leading to a higher prevalence of simulator sickness. Postural instability theory suggests that prolonged postural instability in unfamiliar situations creates simulator sickness [39, 40]. Specifically, the summation of the body’s natural sway and the imposed sway created by movement of the virtual scene may exaggerate postural instability. The reactive control of this instability may enhance symptoms related simulator sickness [39]. Since older adults and individuals with PD have impairments in control of postural stability [41–43], simulator sickness may be exaggerated in these populations. However, this has yet to be investigated.

Furthermore, although a few research studies have successfully used HMDs in individuals with PD for walking in VR, these studies involved only a short period of walking as they were designed to investigate responses to certain manipulation in VR, not potential adverse effects associated with extended exposure. Therefore, time spent in the virtual environment in these studies was much shorter, approximately total 2–3 min based on the distance walked and reported gait speed [44–46]. In constrast, gait training in the clinic often requires bouts of walking of at least 20 min [47]. To date, the potential adverse effects of longer walking experiences in fully immersive VR in older adults and individuals with PD have not been systematically explored.

Thus, the objectives of this study were to determine if older adults and individuals with PD can use fully immersive VR using a head-mounted display for a longer period of walking without adverse effects. To this end, older adults and individuals with PD were exposed to an immersive VR experience using a low-cost HMD (Oculus Rift) during walking on a treadmill. Self-reported (simulator sickness and emotional state) and performance (static and dynamic balance) data were analyzed to determine if there were any adverse effects associated with exposure to the virtual environment. Our results provide needed baseline data to support the potential use of immersive VR using commercial HMDs as a gait training tool for individuals with PD.

## Methods

### Participant characteristics

A total of 33 individuals participated in this study (Table 1) including 11 healthy young adults (HY, 28 ± 7 years, 5 M), 11 healthy older adults (HO, 66 ± 3 years, 3 M), and 11 individuals with Parkinson’s disease PD (65 ± 7 years, 3 M). Participants were included in the study if they were able to walk for 30 min on a treadmill, had a Montreal Cognitive Assessment (MoCA) score between 19 and 30, which corresponds to having no more than mild cognitive impairment [48], and had no other neurological disorders. Participants with PD were classified as modified Hoehn and Yahr (mH&Y) stage 1–3 with an average stage of 2 ± 1, and had Movement Disorder Society Unified Parkinson’s Disease Rating Scale (MDS-UPDRS) part III mean motor scores of 17 ± 8 while on medication (Table 2). Nine of the PD participants consumed their medication upon arrival to the laboratory and two were unable to change their medication schedule. The MoCA score from one participant with PD was lost due to a technical problem, and therefore the reported MoCA scores for the PD group only included 10 participants. Study procedures were approved by the Institutional Review Board at the University of Southern California and all participants provided written, informed consent before testing began. All aspects of the study conformed to the principles described in the Declaration of Helsinki.

**Table 1:** Participant demographics

	HY	HO	PD
Total participants	11	11	11
Age	28 ± 7	66 ± 3	65 ± 7
Gender	5 Male	3 Male	3 Male
MoCA	29 ± 1	27 ± 2	26 ± 3 (for 10 participants)
ABC	96 ± 5	87 ± 11	74 ± 2
10 m walk test (m/s)	1.34 ± 0.19	1.08 ± 0.34	1.16 ± 0.18
Treadmill speed (m/s)	1.22 ± 0.30	1.00 ± 0.30	0.98 ± 0.27

**Table 2:** Characteristics of the participants with PD

Sex	Age	Years since diagnosis	MoCA	MDS-UPDRS (III)	mH&Y	Rigidity^a^			History of FOG^a^	ABC
						Neck	UE^b^	LE^b^		
M	71	32	-	19	2	1	R1	L1	-	94
F	71	10	28	10	1.5	0	0	0	0	84
F	76	9	30	26	1.5	0	0	0	1	89
F	74	3	27	15	3	1	0	R, L1	0	73
F	61	3	29	22	1.5	1	R, L1	0	0	88
F	62	3	28	30	2.5	2	R, L1	0	0	92
M	56	5	26	8	1.5	0	R, L1	0	0	88
F	60	1	20	7	1	0	0	0	1	64
M	69	4	25	14	1.5	0	0	R1	0	53
F	60	2	21	28	3	0	0	0	0	25
F	55	5	26	13	2	0	0	R2, L1	1	66

*UE* Upper extremity, *LE* Lower extremity, *R* Right, *L* Left, *FOG* Freezing of Gait

^a^Score from question 2.13 (Freezing) in the MDS-UPDRS

^b^The first letter represents the side of the limb and second number represents the MDS-UPDRS score

### Experimental protocol

**Fig. 1:**
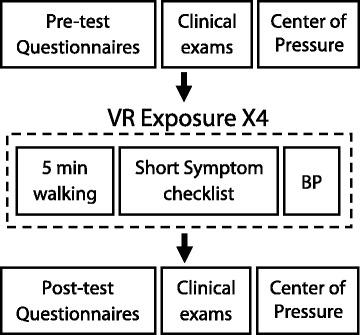
Experimental protocol. Pre-test evaluation involved a set of clinical assessments (MDS-UPDRS for the PD group, mini-BESTest, 10 m walk test, MoCA), static postural stability measures (CoP during eyes open and closed) and questionnaires (SSQ, SAC). The test consisted of walking for a total of 20 min with breaks taken every 5 min for participants to complete a short symptom checklist and for measures of blood pressure (BP) to be monitored. Post-test evaluation involved an additional set of clinical assessments (mini-BESTest, 10 m walk test), static postural stability measures and questionnaires (SSQ, SAC, PQ)

The experimental protocol involved a set of clinical exams, a 20-min walking period, and a set of pre/post evaluations (Fig. 1). For participants with PD, we began by measuring the motor sub-score of the MDS-UPDRS as a measure of motor dysfunction [49]. Next, or first for participants without PD, we measured self-selected walking speed using the 10 m walk test as it is a valid and reliable measure for assessing functional community ambulation in individuals with PD and older adults [50, 51]. We then performed baseline measures of dynamic balance using the Mini-Balance Evaluations Systems Test (Mini-BESTest), a 14-item balance assessment for dynamic balance and gait, which has been shown to be reliable for assessing balance disorders and fall risk in individuals with PD [52]. The Activities-Specific Balance Confidence (ABC) Scale was used to assess each participant’s overall confidence in walking without falling or feeling unsteady [53]. Quantitative assessments of each individual’s level of static postural sway were performed by measuring their center of pressure (CoP) excursion for two, 30 s trials of quiet standing; one trial with their eyes open and one with their eyes closed. Participants were asked to place their feet shoulder-width apart while standing on two force platforms (Bertec, USA), and to stand as still as possible while looking straight ahead. Anteroposterior (AP) and mediolateral (ML) excursions of the CoP were sampled at 1000 Hz.

Baseline levels of symptoms associated with simulator sickness were determined using the Simulator Sickness Questionnaire (SSQ) [54]. The SSQ includes 16 questions related to simulator sickness and, by administering pre- and post-test assessments, allowed us to detect changes in symptoms of nausea, oculomotor discomfort or disorientation due to exposure to the immersive virtual environment. Participants answered each of the 16 questions based on the severity of symptoms they experienced using a 4-point scale from ‘none’ to ‘severe’ (0–3). The point value for each question was summed for corresponding subcategories (Nausea, Oculomotor, and Disorientation), then each subcategory was multiplied by weights of 9.54, 7.58, and 13.92 for Nausea, Oculomotor, and Disorientation, respectively [54]. All subcategories were then summed and multiplied by 3.74 to generate a total score with a possible range of 0–235.6 [54]. A cutoff score of 15 was used to determine if participants experienced significant simulater sickness after exposure as it represents the 75th percentile of scores for a wide range of standard virtual reality simulators [54]. Similarly, cutoff scores of 9.5, 15.2, and 0 were used for the Nausea (N), Oculomotor (O), and Disorientation (D) subscales to determine if individual sensory domains were differentially affected by exposure to the virtual environment [54]. The SSQ has been shown to be reliable in healthy adults (split-half correlation *r* = 0.80) [55] using split-half reliability rather than test-re-test reliability due to potential habituation or adaptation effects across repeated tests.

Lastly, we assessed baseline mood state using the Stress Arousal Checklist (SAC) [56]. The SAC is used to characterize mood, specifically stress and arousal responses, in a variety of situations. Thus, it was used to determine if there were adverse changes in mood due to the VR experience. The SAC contains 33 stress- and arousal-related questions and participants answered each question according to a 4 point scale from ‘definitely do not feel’ to ‘definitely feel’ (1–4) [56]. Each answer was multiplied by an appropriate weight and the values were summed. Scores for stress ranged from −13.65 to 23.4 with higher scores indicating more stress. Scores for arousal ranged from −9.91 to 17.66 with higher values corresponding to increased arousal. The split-half reliability coefficients for both stress and arousal were *r* = 0.80 and 0.82, respectively in healthy adults [57]. The SAC was tested using split-half reliability measures instead of test-re-test metrics because of the transient nature of one’s emotional state [58].

Next, participants walked on the treadmill at the speed that was measured during the 10 m walk test to determine if they were comfortable using this speed for the duration of the treadmill trials. Thirteen participants (3 HY, 5 HO and 5 participants with PD) chose to reduce the speed of the treadmill below their 10 m walk speed either because they were not familiar with walking on a treadmill or because they perceived the speed on the treadmill to be faster than over-ground (Table 1). For all other participants, the speed determined from their 10 m walk was used for all treadmill trials.

### Virtual reality exposure

Participants viewed our immersive virtual environment via a head-mounted display (Oculus Rift Development Kit 2, Oculus VR, LLC) while walking on the treadmill. The display was calibrated to each participant’s measured inter-pupillary distance and horizontal field of view using a calibration sequence provided by Oculus. After calibration, participants donned the HMD and walked for four, 5 min bouts on the treadmill (Bertec Fully Instrumented Treadmill, USA). After each 5-min bout, participants were given a 1-min rest break during which they completed a Short Symptom Checklist [59] to report any symptoms of simulator sickness. The checklist is a 6-item questionnaire adapted from the SSQ [59]. Blood pressure and heart rate were also measured during the rest break.

**Fig. 2:**
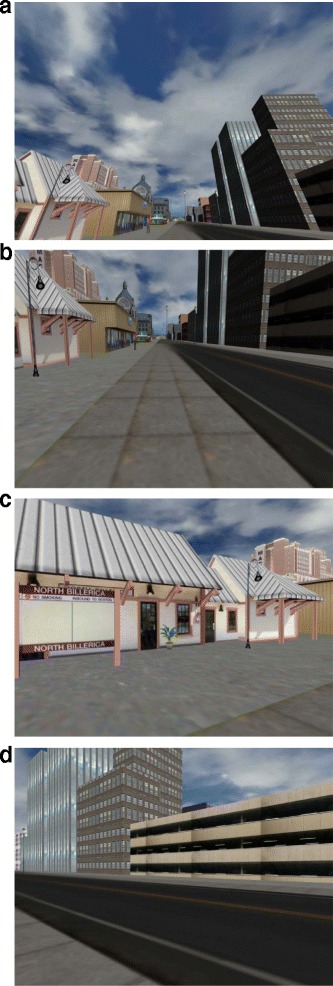
Representative images of the virtual environment. The environment consisted of a cityscape including buildings, avatars and a 800 m pedestrian path. A first person views (**a**) up, (**b**) front, (**c**) left, and (**d**) right

The virtual environment shown on the HMD was developed using Sketchup (Trimble Navigation Limited, USA), and the participants’ interaction with the environment was controlled using Vizard (WorldViz, USA). The virtual environment consisted of a cityscape with buildings, animated avatars, and an 800 m straight sidewalk (Fig. 2). The avatars were added to provide a dynamic element to the virtual environment, and they were the only animated features of the environment. Movements through the environment were constrained to the forward direction, but participants were able to freely look around the scene while walking. The environment did not include any turns, doorways, or crossing of thresholds. The velocity of the simulation was synchronized to the speed of the treadmill, and the orientation of the participants’ viewpoint was synchronized with head orientation using an inertial measurement unit embedded in the HMD. The environment was updated at refresh rate ~60 frames per second and the simulation was run on a desktop computer with 8 GB RAM, an Intel Core i7 CPU, and an NVIDIA GeForce GTX 770 GPU. The HMD had a resolution of 960 × 1080 pixels for each eye, a refresh rate of up to 75 Hz, a 100° field of view, and a mass of approximately 450 g.

### Post-test assessments

Following the four walking bouts, participants were re-assessed using the 10 m walk test, mini-BESTest, and measures of CoP excursion to determine if exposure to the virtual enevironment resulted in any motor disturbances. Participants also completed a series of post-test questionnaires including the SSQ, SAC, and the Presence questionnaire (PQ, version 2.0). The PQ was used to measure subjective experience of being in a virtual environment, or “presence” [24]. The PQ contained 19 virtual experience-related questions (e.g. ‘How closely were you able to examine objects in the virtual environment?’). Participants described their degree of presence using a scale from ‘not at all’ to ‘extremely’ (1–7). The answers were summed yielding a possible range of 19–133. Subscales of the PQ included the following: Involvement/Control (score range: 10–70); Visual fidelity (2–14); Adaptation/Immersion (4–28); and Interface quality (3–21) [60]. The reliability of the PQ in healthy young adults was established using internal consistency measures of reliability (Cronbach’s Alpha), which yielded an alpha of 0.88 [24].

### Data analysis

Mean sway area was computed from the COP data in Matlab (Natick, MA) using the method described in Duarte and Zatsiorsky [61]. Briefly, the net CoP was computed from a weighted average of the individual CoPs from each force platform. The net CoP trajectory was then fit with a 95% confidence ellipse, and the area of this ellipse was used to quantify sway area.

### Statistical analyses

All statistical analyses were performed in Matlab. A two-way, repeated measures ANOVA was performed to test for an effect of group (HY, HO, or PD) or time (Pre- and Post-test) on the SSQ, SAC, mini-BESTest, and 10 m walk. A three-way, repeated measures ANOVA was performed to test for an effect of group, time, or vision condition (EO and EC) on CoP sway area. A one-way ANOVA was performed to test for an effect of group on the PQ. Significance was assessed at the *p* < 0.05 level. If there were significant main effects or interactions, post hoc comparisons were performed using Tukey’s Honest Significant Difference.

## Results

### Simulator sickness

**Fig. 3:**
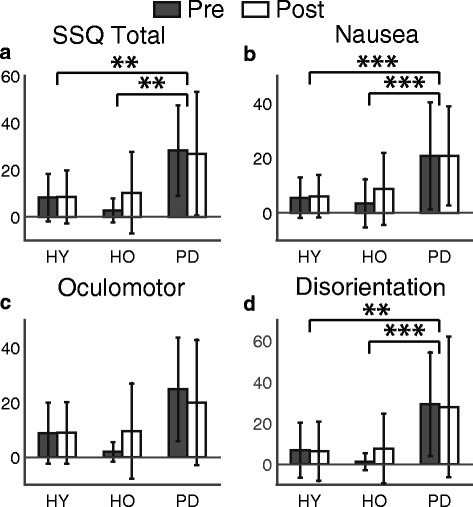
Pre- (*dark gray bar*) and post-test (*white bar*) average simulator sickness questionnaire total score and sub-category scores with standard deviation. *Asterisks* represent statistical significance (**: *p* < 0.01, ***: *p* < 0.005). **a** Average total score of SSQ. **b** Average Nausea score. **c** Average Oculomotor discomfort score. **d** Average Disorientation score

Simulator-related sickness symptoms were not enhanced after the VR exposure in any groups (Fig. 3). There was a significant main effect of group (F(2, 30) = 6.05, *p* < 0.01) on the total simulator sickness score, but no effect of time (F(1, 30) = 0.447, *p* = 0.51) or interaction between group and time (F(2, 30) = 0.691, *p* = 0.51). Post hoc analysis revealed that the PD group presented higher SSQ scores overall compared to HY (*p* < 0.01) and HO groups (*p* < 0.01). The average SSQ scores were 8.3 ± 10.5, 6.5 ± 13.0, and 27.5 ± 22.5 for HY, HO, and PD groups, respectively. All participants completed four bouts of walking and no participants verbally reported any symptoms of simulator sickness. Overall, the individual analysis of score changes between pre- and post-test showed that two participants from the HO group and one participant from PD group had post-test scores that were at least 15 points higher than their pre-test scores, which is a cut-off score of having simulator sickness [54]. This suggests that there may be a small fraction of these populations who may not be good candidates for locomotor training in VR.

Analysis of the sub-scores of the SSQ indicated that no specific domains of simulation-related sickness were changed by VR exposure. There was a main effect of group (F(2,30) = 7.75, *p* < 0.01) on the level of nausea (N), but no effect of time (F(1,30) = 0.328, *p* = 0.57) nor interaction between time and group (F(2,30) = 0.245, *p* = 0.78). Post hoc analysis showed that the PD group had higher scores than HY (*p* < 0.01) and HO group (*p* < 0.01). The average N scores were 5.8 ± 7.4, 6.1 ± 11.2, and 20.8 ± 18.3 for HY, HO, and PD groups, respectively. Individual analysis showed that 3 participants in each group increased their N scores above the cutoff of 9.5. There were no main effects of group (F(2,30) = 2.93, *p* = 0.069), time (F(1.30) = 0.223, *p* = 0.64), nor interaction between time and group (F(2,30) = 3.10, *p* = 0.06) on oculomotor (O) scores, and only 2 HO participants increased the O scores above 15.2. There was a main effect of group (F(2,30) = 5.87, *p* < 0.01) on disorientation (D) scores, but no effects of time (F(1,30) = 0.116, *p* = 0.74) or interaction between time and group (F(2,30) = 0.283, *p* = 0.76). Post hoc analysis showed that the score of PD group was higher than HY group (*p* < 0.01) and HO group (*p* < 0.01). The average D scores were 6.5 ± 13.6, 4.4 ± 12.4, and 28.5 ± 29.3 for HY, HO, and PD groups, respectively. Individual analysis revealed that 1 HY, 3 HO and 3 PD increased the D scores above 0.

We found no main effects of group (F(2, 30) = 0.892, *p* = 0.42) or time (F(2, 60) = 0.353, *p* = 0.70) or interaction between group and time (F(4, 60) = 0.823, *p* = 0.52) for the short symptom checklist. This indicates that all groups rapidly acclimated to our virtual environment.

### Center of pressure (CoP)

**Fig. 4:**
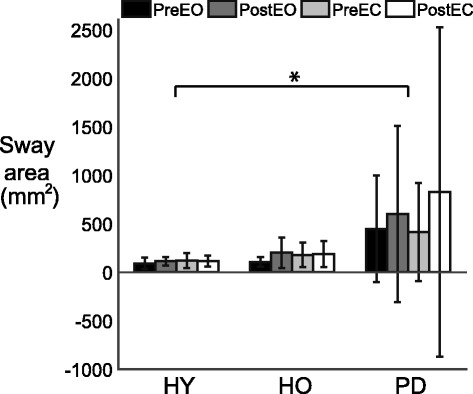
CoP sway area. Pre- (*black bar*) and post-test (*dark gray bar*) during eyes open (EO) and pre- (*light gray bar*) and post-test (*white bar*) during eyes closed (EC). Vertical bars represent standard deviations. Asterisks represent statistical significance (*: *p* < 0.05)

Postural sway, as measured by CoP area, was not affected by VR exposure in any of our groups (Fig. 4). There was a significant main effect of group (F(2,60) = 6.51, *p* < 0.01) on sway area, but no effect of time (F(1,60) = 1.47, *p* = 0.23), vision condition (F(1,60) = 0.178, *p* = 0.68) nor interaction between group and time (F(2,60) = 0.809), *p* = 0.45), between time and condition (F(1,60) = 0.0735, *p* = 0.79), between group and condition (F(2,60) = 0.050, *p* = 0.95), or among group, time and condition (F(2,60) = 0.319, *p* = 0.73). Specifically, post-hoc analyses revealed that the sway area in the PD group was greater than the sway area in the HY group (*p* < 0.05). Average sway areas were 109 ± 60, 168 ± 125, and 572 ± 1010 mm^2^ for HY, HO and PD, respectively. The data indicate that static postural control measured by CoP sway was not affected by the VR exposure.

### Clinical assessment

Interestingly, both mini-BESTest scores and self-selected gait speed improved after VR exposure. There were significant differences of group (F(2,30) = 11.0, *p* < 0.001) and time (F(1,30) = 7.58, *p* < 0.01) on mini-BESTest scores, but no significant interaction between group and time (F(2,30) = 1.96, *p* = 0.16). Post hoc analysis showed PD (*p* < 0.001) and HO (*p* < 0.01) groups had lower mini-BESTest scores than the HY group. Moreover, the post-performance improved compared to the pre-performance. The average mini-BESTest scores were 28 ± 1, 23 ± 4, and 21 ± 4 for pre-test HY, HO, and PD groups and 28 ± 1, 25 ± 3, and 23 ± 4 for post-test HY, HO, and PD groups, respectively. We found a significant effect of group (F(2,30) = 5.33, *p* < 0.05) and time (F(1, 30) = 5.99, *p* < 0.05) on gait speed, but no significant interaction (F(2,30) = 0.60, *p* = 0.55). The speed of the HY group was significantly faster than the HO group (*p* < 0.05) and, across all groups, participants walked significantly faster after exposure, where baseline speed was 1.34 ± 0.19 m/s, 1.08 ± 0.34 m/s, and 1.16 ± 0.18 m/s for HY, HO, and PD, respectively and post-exposure speed was 1.42 ± 0.17 m/s, 1.12 ± 0.27 m/s, and 1.20 ± 0.18 m/s for HY, HO, and PD, respectively.

### Stress-arousal

We found significant effects of exposure to the virtual environment on both stress and arousal. For the level of stress, all groups presented less stress after exposure to the environment. The stress level was significantly affected by time (F(1,30) = 7.07, *p* < 0.05), but not affected by group (F(2,30) = 1.08, *p* = 0.35), and there was no interaction between group and time (F(2,30) = 3.13, *p* = 0.058). Post hoc analysis of time effect showed that post-stress score was significantly lower than pre-stress score, where the total stress scores were −9 ± 4 and −11 ± 3 for pre- and post-test, respectively. For the level of arousal, the PD group showed a larger increase after exposure relative to either of the healthy groups. There were no main effects of time (F(1,30) = 2.30, *p* = 0.14) or group (F(2,30) = 0.175, *p* = 0.84) on arousal scores, but there was a significant interaction between group and time (F(2,30) = 4.67, *p* < 0.05). Post hoc analysis for the interaction between group and time showed that the score change between pre- and post-exposure was greater in the PD group compared to the HY and HO groups. The absolute score changes of the level of arousal were 3 ± 2, 4 ± 4, 8 ± 7 for HY, HO, and PD groups, respectively.

### Presence

**Fig. 5:**
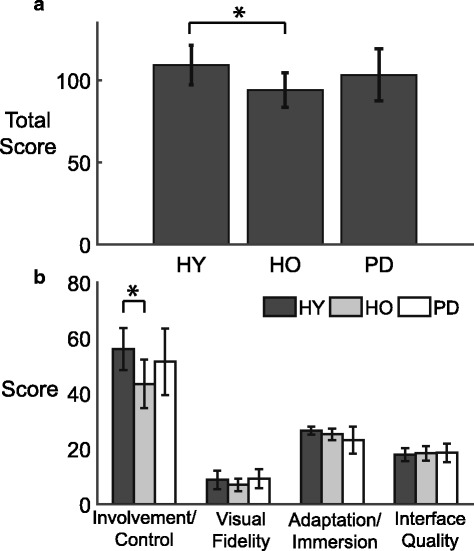
Average presence questionnaire scores are shown with individual scores. **a** Average total score. **b** Average sub-category scores; Involvement/Control, visual fidelity, adaptation/Immersion and interface quality for HY (*dark gray bar*), HO (*light gray bar*), and PD (*white bar*). Vertical bars represent standard deviations. Asterisks represent statistical significance (*: *p* < 0.05)

Healthy young adults experienced a higher sense of presence in the virtual space than healthy older adults (Fig. 5). There was a main effect of group on the level of perceived presence in the virtual environment (F(2,30) = 3.77, *p* < 0.05). Specifically, the HO group had significantly lower scores than the HY group (*p* < 0.05). When examining the sub-categories of the PQ, we found a main effect of group on the Involvement/Control score (F(2,30) = 4.79, *p* < 0.05), with the HY score being higher than HO (*p* < 0.05). This higher Involvement/Control score in the HY group relative to the HO groups is the likely source of the observed differences in overall presence. Lastly, there were no effects of group on Visual Fidelity (F(2,30) = 1.61, *p* = 0.22), Adaptation/Immersion (F(2,30) = 3.19, *p* = 0.056), or Interface Quality (F(2,30) = 0.149, *p* = 0.86). The presence score for the PD group was not significantly different from either of the healthy groups.

## Discussion

The aim of this study was to evaluate if age or Parkinson’s disease affects the safety of using immersive VR with a head-mounted display for extended periods of walking. Despite emerging uses of VR in the context of gait rehabilitation for individuals with PD [9, 13, 16], previous studies have not explicitly investigated potential negative aftereffects of using fully immersive VR. Our study revealed that symptoms of simulator-related sickness and static and dynamic postural control were not affected by exposure to a fully immersive environment for the majority of participants. Thus, older adults and individuals with mild to moderate PD are capable of using immersive VR for a total of 20 min of walking practice in 5-min bouts with minimal negative aftereffects.

Simulator sickness is one of the most common side effects of using VR, but we found that our virtual simulation elicited only minor changes in symptoms of nausea, oculomotor discomfort, or disorientation. Simulator sickness is an undesirable side effect of VR experiences when using HMDs for flight simulations or 3D video games [27–29], and is also an unwanted side effect for VR-based rehabilitation interventions. We found that overall SSQ scores were higher in our PD group, but this is likely a side-effect of medication [62] or an expression of non-motor symptoms of PD such as nausea [63] given that these symptoms were present before exposure to the virtual environment. The majority of participants in all of our groups successfully completed a total of 20 min of walking in the virtual environment without increasing the severity of simulator sickness. This indicates that age and the presence of PD have negligible influence on the feasibility of using fully immersive virtual reality with recent, commercial HMDs.

Postural disequilibrium is another common side effect of VR [64], but this was not observed in any of our test groups. Overall, larger and more variable sway area during quiet standing was observed in the PD group relative to the HY and HO groups. This indicates that static postural stability was inherently lower in the PD group in agreement with previous work [41, 42]. The HO and PD groups had lower mini-BESTest scores than the HY group indicating that the HO and PD groups were more dynamically unstable than the HY group due to the effects of age [65] and/or Parkinson’s disease [66]. However, neither sway area nor mini-BESTest scores was adversely affected by exposure to the current simulation. Interestingly, we observed a significant increase in the mini-BESTest scores after VR exposure, suggesting that dynamic postural control may have improved. However, since the increase in the score did not reach the minimal detectable change (MDC of mini-BEST for PD = 5.52 [67]), this improvement may not have been meaningful. In summary, these findings indicate that postural disequilibrium is not a concern after walking in our simulation.

An alternative hypothesis for the lack of negative effects in our PD group is that any sensory deficits present in this group may have caused them to be less prone to sensory mismatch-induced simulator sickness. In other words, if there is incongruence between visual, vestibular, and proprioceptive information, the resulting mismatch signal may be underweighted and therefore have little influence on perceptions of postural stability. One way to determine the likelihood of this explanation would be to compare the effects of an imposed sensory mismatch on symptoms of simulator sickness in individuals with PD who have known sensory deficits. If these individuals prove to be less prone to symptoms of simulator sickness than healthy controls, then this would provide support for the hypothesis that sensory mismatches are down-weighted in this population.

We also found that individuals with PD were more emotionally aroused post-exposure compared to HY and HO groups, and participants in all groups were less stressed after VR exposure. It is well-established that the level of arousal increases immediately after aerobic exercise in healthy younger adults [68–70] and healthy older women [71]. This increase in the level of arousal may also have occurred in the PD group due to the exertion associated with the clinical assessments and subsequent walking bouts. Alternatively, medication may have played a role in the increased levels of arousal. The pre-SAC was completed immediately after medication was consumed and the post-SAC was completed after approximately 2 h. Therefore, it is quite possible that medication played a role in the increased level of arousal post-test. The decreased stress level observed after VR exposure may result from the participants initially feeling stressed and anxious about the unknown experience involved with virtual reality. Then, as the VR exposure progressed, they may have become more relaxed after acclimating to the system. Given these results, we believe that it is important for future studies to evaluate the potential for VR and game-based interventions to improve aspects of mood and psychological state in addition to changes in physical performance.

### Relevance to proposed theories of simulator sickness

Our results also provide some insight into the robustness of previously proposed theories of simulator-related sickness. Although individuals with PD had significantly higher sway area and lower mini-BESTest scores than age-matched controls, this did not increase their likelikehood of simulator sickness. This suggests that the postural instability theory is not a sufficient explanation of the cause of simulator sickness as this theory would predict that simulator sickness should scale with postural instability. In contrast, our results provide little evidence regarding the potential contribution of the sensory conflict theory to simulator sickness. The visual scene in our simulation was designed to be congruent with the user’s locomotor behavior as the speed of translation through the simulated environment was matched to the participants’ walking speed. This congruency may have minimized simulator-related sickness and would be consistent with the work of Jaeger and Mourant who found that dynamic walking in VR reduces simulator-related symptoms compared to a static simulation in younger adults [72]. However, it is important to note that we did not measure possible deficits in sensory integration for our participants. Therefore, it may be possible that they simply did not have sufficient deficits in sensory integration to generate simulator sickness via sensory conflict.

With recent technological advances, the availability and practicality of hardware and software for VR-based interventions has improved significantly compared to even a few years ago. The virtual simulation used in this study was able to maintain framerates of approximately 60 frames per second, had a wide field of view, and was capable of responding to changes in head roll, pitch, and yaw. In contrast, previous studies that used immersive VR with HMDs reported that they maintained approximately 20–40 frames per second, which can induce high delay and lag between the movement and the simulation [29]. This may have caused significant simulator sickness in those studies and would likely interfere with the immersive experience. Thus, if a virtual simulation can maintain high update and tracking rates, the occurrence of side effects from using VR can be minimized.

### Study limitations

There were few limitations in this study. First, to better generalize our results, future studies including a larger range of disease severity and a larger sample size that is more representative of the PD population are needed. Our study included more women than men in the HO and PD groups, and considering the higher prevalence of PD in men [73], future studies should reflect this prevalence bias in their study sample. Second, the questionnaires used in our study have only been validated in healthy adults, and therefore further research is warranted to establish the psychometric properties of these questionnaires in individuals with PD. Likewise, the SSQ cutoff score that we used to establish a threshold for simulator sickness has only been established in healthy younger adults. We expected to see higher SSQ scores in older adults and individuals with PD due to the possibility that they might have an impaired ability to resolve sensory conflicts and maintain postural stability. However, we found that only a few participants had SSQ scores higher than 15, and no participants verbally indicated that they had symptoms of simulator sickness. Nevertheless, future studies should establish an appropriate SSQ cutoff score for older adults and individuals with PD.

We should also note that our VR task was not specifically designed to challenge the known gait-related sensorimotor deficits of individuals with Parkinson’s disease, and as a result, this may have contributed to the lack of adverse effects observed in our study. The performance of more challenging walking tasks such as turning, obstacle negotiation, or passing through doorways is known to be impaired in individuals with PD [9, 74, 75]. It remains to be seen if these impairments, when combined with performance of similar tasks in a virtual environment, influence the likelihood of observing simulator-related sickness symptoms. Moreover, since practicing these types of walking tasks are likely to have the greatest clinical utility, future studies should continue to carefully investigate the safety and feasibility of using fully immersive VR for clinical gait training.

## Conclusions

In summary, the aim of our study was to determine if age or pathology play a role in the observation of negative physiological and psychological effects after using immersive VR. We found that 20 min of walking in an immersive environment using the Oculus Rift did not induce simulator-related sickness, or alterations in static or dynamic postural control. This provides a foundation for future studies exploring novel and innovative approaches to simulate real-world challenges using immersive virtual reality while increasing immersion during gait training.

## Abbreviations

ABC: Activities-Specific Balance Confidence
BESTest: Balance Evaluations Systems Test
CoP: Center of pressure
D: Disorientation
DRT: Dopamine replacement therapy
FOG: Freezing of Gait
HMD: Head-mounted display
HO: Healthy older
HY: Healthy young
LE: Lower extremity
MDS-UPDRS: Movement Disorder Society Unified Parkinson’s Disease Rating Scale
mH&Y: Modified Hoehn and Yahr
N: Nausea
O: Oculomotor
PD: Parkinson’s disease
PQ: Presence questionnaire
SAC: Stress Arousal Checklist
SSQ: Simulator sickness questionnaire
UE: Upper extremity
VR: Virtual reality

## References

[1] Ross GW, Abbott RD (2014). Living and dying with Parkinson’s disease. Mov Disord.

[2] Weintraub D, Comella CL, Horn S (2008). Parkinson’s disease—Part 1: Pathophysiology, symptoms, burden, diagnosis, and assessment. Am J Manag Care.

[3] Grimbergen YAM, Munneke M, Bloem BR (2004). Falls in Parkinson’s disease. Curr Opin Neurol.

[4] Grabli D, Karachi C, Welter M-L, Lau B, Hirsch EC, Vidailhet M, et al. Normal and pathological gait: what we learn from Parkinson’s disease. J Neurol Neurosurg Psychiatry. 2012. doi:10.1136/jnnp-2012-302263. 10.1136/jnnp-2012-302263 PMC3852420 22752693

[5] Kleim JA, Jones TA (2008). Principles of experience-dependent neural plasticity: implications for rehabilitation after brain damage. J Speech Lang Hear Res.

[6] Petzinger GM, Fisher BE, Van Leeuwen J-E, Vukovic M, Akopian G, Meshul CK (2010). Enhancing neuroplasticity in the basal ganglia: the role of exercise in Parkinson’s disease. Mov Disord.

[7] Herman T, Giladi N, Gruendlinger L, Hausdorff JM (2007). Six Weeks of Intensive Treadmill Training Improves Gait and Quality of Life in Patients With Parkinson’s Disease: A Pilot Study. Arch Phys Med Rehabil.

[8] de Goede CJT, Keus SHJ, Kwakkel G, Wagenaar RC (2001). The effects of physical therapy in Parkinson’s Disease: A research synthesis. Arch Phys Med Rehabil.

[9] Mirelman A, Maidan I, Herman T, Deutsch JE, Giladi N, Hausdorff JM (2011). Virtual reality for gait training: can it induce motor learning to enhance complex walking and reduce fall risk in patients with Parkinson’s disease?. J Gerontol A Biol Sci Med Sci.

[10] Yang Y-R, Tsai M-P, Chuang T-Y, Sung W-H, Wang R-Y (2008). Virtual reality-based training improves community ambulation in individuals with stroke: a randomized controlled trial. Gait Posture.

[11] Jaffe DL, Brown DA, Pierson-Carey CD, Buckley EL, Lew HL (2004). Stepping over obstacles to improve walking in individuals with poststroke hemiplegia. J Rehabil Res Dev.

[12] Parijat P, Lockhart TE, Liu J (2015). Effects of perturbation-based slip training using a virtual reality environment on slip-induced falls. Ann Biomed Eng.

[13] Shema S, Brozgol M, Dorfman M, Maidan I, Yannai OW, Giladi N (2013). Clinical experience using a 5 week treadmill training program with virtual reality to enhance gait. 2013 International Conference on Virtual Rehabilitation (ICVR).

[14] Fung J, Richards CL, Malouin F, McFadyen BJ, Lamontagne A (2006). A treadmill and motion coupled virtual reality system for gait training post-stroke. Cycberpsychol Behav.

[15] Rizzo A, Kim GJ (2005). A SWOT Analysis of the Field of Virtual Reality Rehabilitation and Therapy. Presence Teleop Virt.

[16] Mirelman A, Rochester L, Reelick M, Nieuwhof F, Pelosin E, Abbruzzese G (2013). V-TIME: a treadmill training program augmented by virtual reality to decrease fall risk in older adults: study design of a randomized controlled trial. BMC Neurol.

[17] Mirelman A, Rochester L, Maidan I, Del Din S, Alcock L, Nieuwhof F, et al. Addition of a non-immersive virtual reality component to treadmill training to reduce fall risk in older adults (V-TIME): a randomised controlled trial. Lancet. 2016. doi:10.1016/S0140-6736(16)31325-3. 10.1016/S0140-6736(16)31325-3 27524393

[18] Cruz-Neira C, Sandin DJ, DeFanti TA, Kenyon RV, Hart JC (1992). The CAVE: Audio Visual Experience Automatic Virtual Environment. Commun ACM.

[19] Cruz-Neira C, Sandin DJ, DeFanti TA (1993). Surround-screen Projection-based Virtual Reality: The Design and Implementation of the CAVE. Proceedings of the 20th Annual Conference on Computer Graphics and Interactive Techniques.

[20] Denisova A, Cairns P (2015). First Person vs. Third Person Perspective in Digital Games: Do Player Preferences Affect Immersion?. Proceedings of the 33rd Annual ACM Conference on Human Factors in Computing Systems.

[21] Kozhevnikov M, Dhond RP. Understanding Immersivity: Image Generation and Transformation Processes in 3D Immersive Environments. Front Psychol. 2012;3. doi:10.3389/fpsyg.2012.00284. 10.3389/fpsyg.2012.00284 PMC3415688 22908003

[22] Riva G, Davide F, IJsselsteijn WA (2003). Being There: Concepts, Effects and Measurements of User Presence in Synthetic Environments.

[23] Rand D, Kizony R, Feintuch U, Katz N, Josman N, Rizzo A, Weiss PL. Comparison of Two VR Platforms for Rehabilitation: Video Capture versus HMD. Presence Teleop Virt. 2005;14:147–60. doi:10.1162/1054746053967012.

[24] Witmer BG, Singer MJ (1998). Measuring Presence in Virtual Environments: A Presence Questionnaire. Presence Teleop Virt.

[25] Schultheis MT, Rizzo AA (2001). The application of virtual reality technology in rehabilitation. Rehabil Psychol.

[26] Bailenson J, Patel K, Nielsen A, Bajscy R, Jung S-H, Kurillo G (2008). The Effect of Interactivity on Learning Physical Actions in Virtual Reality. Med Psychol.

[27] Kennedy RS, Lilienthal MG, Berbaum KS, Baltzley DR, McCauley ME (1989). Simulator sickness in U.S. Navy flight simulators. Aviat Space Environ Med.

[28] Regan EC, Price KR (1994). The frequency of occurrence and severity of side-effects of immersion virtual reality. Aviat Space Environ Med.

[29] Sharples S, Cobb S, Moody A, Wilson JR (2008). Virtual reality induced symptoms and effects (VRISE): Comparison of head mounted display (HMD), desktop and projection display systems. Displays.

[30] Treleaven J, Battershill J, Cole D, Fadelli C, Freestone S, Lang K, et al. Simulator sickness incidence and susceptibility during neck motion-controlled virtual reality tasks. Virtual Reality. 2015;1–9. doi:10.1007/s10055-015-0266-4.

[31] Young MK, Gaylor GB, Andrus SM, Bodenheimer B (2014). A Comparison of Two Cost-differentiated Virtual Reality Systems for Perception and Action Tasks. Proceedings of the ACM Symposium on Applied Perception.

[32] Johnson DM. Introduction to and review of simulator sickness research. Fort Rucker: Rotary-Wing Aviation Research Unit, U.S. Army Research Institute for the Behavioral and Social Sciences; 2005.

[33] Reason J, Brand JJ, Reason JT, Brand JJ (1975). Motion sickness.

[34] Reason JT (1978). Motion sickness adaptation: a neural mismatch model. J R Soc Med.

[35] Brooks JO, Goodenough RR, Crisler MC, Klein ND, Alley RL, Koon BL (2010). Simulator sickness during driving simulation studies. Accid Anal Prev.

[36] Patel N, Jankovic J, Hallett M (2014). Sensory aspects of movement disorders. Lancet Neurol.

[37] Hwang S, Agada P, Grill S, Kiemel T, Jeka JJ (2016). A central processing sensory deficit with Parkinson’s disease. Exp Brain Res.

[38] Boecker H, Ceballos-Baumann A, Bartenstein P, Weindl A, Siebner HR, Fassbender T (1999). Sensory processing in Parkinson’s and Huntington’s disease. Brain.

[39] Stoffregen TA, Smart LJ (1998). Postural instability precedes motion sickness. Brain Res Bull.

[40] Stoffregen TA, Hettinger LJ, Haas MW, Roe MM, Smart LJ (2000). Postural Instability and Motion Sickness in a Fixed-Base Flight Simulator. Hum Factors.

[41] Schmit JM, Riley MA, Dalvi A, Sahay A, Shear PK, Shockley KD (2006). Deterministic center of pressure patterns characterize postural instability in Parkinson’s disease. Exp Brain Res.

[42] Błaszczyk JW, Orawiec R, Duda-Kłodowska D, Opala G (2007). Assessment of postural instability in patients with Parkinson’s disease. Exp Brain Res.

[43] Benatru I, Vaugoyeau M, Azulay J-P (2008). Postural disorders in Parkinson’s disease. Neurophysiol Clin.

[44] Ehgoetz Martens KA, Ellard CG, Almeida QJ (2015). Does manipulating the speed of visual flow in virtual reality change distance estimation while walking in Parkinson’s disease?. Exp Brain Res.

[45] Ehgoetz Martens KA, Ellard CG, Almeida QJ (2015). Virtually-induced threat in Parkinson’s: Dopaminergic interactions between anxiety and sensory-perceptual processing while walking. Neuropsychologia.

[46] Ehgoetz Martens KA, Ellard CG, Almeida QJ (2015). Anxiety-provoked gait changes are selectively dopa-responsive in Parkinson’s disease. Eur J Neurosci.

[47] Mehrholz J, Friis R, Kugler J, Twork S, Storch A, Pohl M. Treadmill training for patients with Parkinson’s disease. Cochrane Database Syst Rev. 2010;CD007830. doi:10.1002/14651858.CD007830.pub2. 10.1002/14651858.CD007830.pub2 20091652

[48] Nasreddine ZS, Phillips NA, Bédirian V, Charbonneau S, Whitehead V, Collin I (2005). The Montreal Cognitive Assessment, MoCA: a brief screening tool for mild cognitive impairment. J Am Geriatr Soc.

[49] Goetz CG, Tilley BC, Shaftman SR, Stebbins GT, Fahn S, Martinez-Martin P (2008). Movement Disorder Society-sponsored revision of the Unified Parkinson’s Disease Rating Scale (MDS-UPDRS): Scale presentation and clinimetric testing results. Mov Disord.

[50] Bohannon RW (1997). Comfortable and maximum walking speed of adults aged 20–79 years: reference values and determinants. Age Ageing.

[51] Steffen T, Seney M (2008). Test-retest reliability and minimal detectable change on balance and ambulation tests, the 36-item short-form health survey, and the unified Parkinson disease rating scale in people with parkinsonism. Phys Ther.

[52] Godi M, Franchignoni F, Caligari M, Giordano A, Turcato AM, Nardone A (2013). Comparison of reliability, validity, and responsiveness of the mini-BESTest and Berg Balance Scale in patients with balance disorders. Phys Ther.

[53] Lajoie Y, Gallagher SP (2004). Predicting falls within the elderly community: comparison of postural sway, reaction time, the Berg balance scale and the Activities-specific Balance Confidence (ABC) scale for comparing fallers and non-fallers. Arch Gerontol Geriatr.

[54] Robert S, Kennedy NEL (1993). Simulator Sickness Questionnaire: An Enhanced Method for Quantifying Simulator Sickness. Int J Aviat Psychol.

[55] Kennedy RS, Drexler JM, Compton DE, Stanney KM, Lanham S, Harm DL. Configural Scoring of Simulator Sickness, Cybersickness and Space Adaptation Syndrome: Similarities and Differences? [Internet]. 2001. Available: http://ntrs.nasa.gov/search.jsp?R=20100033371. Accessed 16 Feb 2017.

[56] Cox T, Mackay C (1985). The measurement of self-reported stress and arousal. Br J Psychol.

[57] Watts C, Cox T, Robson J (1983). Morningness-Eveningness and Diurnal Variations in Self-Reported Mood. J Psychol.

[58] Wilson JR, Corlett N. Evaluation of Human Work. 3rd ed. Boca Raton: CRC Press; 2005.

[59] Cobb SVG, Nichols S, Ramsey A, Wilson JR (1999). Virtual Reality-Induced Symptoms and Effects (VRISE). Presence Teleop Virt.

[60] Witmer BG, Jerome CJ, Singer MJ (2005). The Factor Structure of the Presence Questionnaire. Presence Teleop Virt.

[61] Duarte M, Zatsiorsky VM (2002). Effects of body lean and visual information on the equilibrium maintenance during stance. Exp Brain Res.

[62] Chaudhuri KR, Schapira AH (2009). Non-motor symptoms of Parkinson’s disease: dopaminergic pathophysiology and treatment. Lancet Neurol.

[63] Chaudhuri KR, Healy DG, Schapira AH (2006). Non-motor symptoms of Parkinson’s disease: diagnosis and management. Lancet Neurol.

[64] Kennedy RS, Stanney KM (1996). Postural instability induced by virtual reality exposure: Development of a certification protocol. Int J Hum Comput Interact.

[65] O’Hoski S, Winship B, Herridge L, Agha T, Brooks D, Beauchamp MK (2014). Increasing the clinical utility of the BESTest, mini-BESTest, and brief-BESTest: normative values in Canadian adults who are healthy and aged 50 years or older. Phys Ther.

[66] Duncan RP, Earhart GM (2012). Should One Measure Balance or Gait to Best Predict Falls among People with Parkinson Disease?. Parkinsons Dis.

[67] Leddy AL, Crowner BE, Earhart GM (2011). Utility of the Mini-BESTest, BESTest, and BESTest sections for balance assessments in individuals with Parkinson disease. J Neurol Phys Ther.

[68] Roth DL, Bachtler SD, Fillingim RB (1990). Acute emotional and cardiovascular effects of stressful mental work during aerobic exercise. Psychophysiology.

[69] Steptoe A, Cox S (1988). Acute effects of aerobic exercise on mood. Health Psychol.

[70] Hoffman MD, Hoffman DR (2008). Exercisers achieve greater acute exercise-induced mood enhancement than nonexercisers. Arch Phys Med Rehabil.

[71] Pierce EF, Pate DW (1994). Mood alterations in older adults following acute exercise. Percept Mot Skills.

[72] Jaeger BK, Mourant RR (2001). Comparison of Simulator Sickness Using Static and Dynamic Walking Simulators. Proc Hum Factors Ergon Soc Annu Meet.

[73] de Lau LM, Breteler MM (2006). Epidemiology of Parkinson’s disease. Lancet Neurol.

[74] Morris ME, Huxham F, McGinley J, Dodd K, Iansek R (2001). The biomechanics and motor control of gait in Parkinson disease. Clin Biomech.

[75] Cowie D, Limousin P, Peters A, Day BL (2010). Insights into the neural control of locomotion from walking through doorways in Parkinson’s disease. Neuropsychologia.

